# Cellular membrane protein MipA from *E.coli* Nissle 1917 protects against *Salmonella* infection

**DOI:** 10.1093/ismejo/wrag054

**Published:** 2026-03-13

**Authors:** Yunqing Xiang, Huipai Peng, Yanxia Li, Hui Wen, Nan Li, Xiaozhi Liu, Guodong Yuan, Yong Shi, Shuqiang Huang

**Affiliations:** State Key Laboratory of Quantitative Synthetic Biology, Shenzhen Institute of Synthetic Biology, Shenzhen Institutes of Advanced Technology, Chinese Academy of Sciences, Shenzhen, Guangdong 518055, China; Department of Mechanical, Materials and Manufacturing Engineering, University of Nottingham Ningbo China, Ningbo 315100, China; State Key Laboratory of Quantitative Synthetic Biology, Shenzhen Institute of Synthetic Biology, Shenzhen Institutes of Advanced Technology, Chinese Academy of Sciences, Shenzhen, Guangdong 518055, China; Tianjin Key Laboratory of Epigenetics for Organ Development of Premature Infants, Tianjin Fifth Central Hospital, Tianjin 300450, China; State Key Laboratory of Quantitative Synthetic Biology, Shenzhen Institute of Synthetic Biology, Shenzhen Institutes of Advanced Technology, Chinese Academy of Sciences, Shenzhen, Guangdong 518055, China; State Key Laboratory of Quantitative Synthetic Biology, Shenzhen Institute of Synthetic Biology, Shenzhen Institutes of Advanced Technology, Chinese Academy of Sciences, Shenzhen, Guangdong 518055, China; Tianjin Key Laboratory of Epigenetics for Organ Development of Premature Infants, Tianjin Fifth Central Hospital, Tianjin 300450, China; State Key Laboratory of Superlattices and Microstructures, Institute of Semiconductors, Chinese Academy of Sciences, Beijing 100083, China; Department of Mechanical, Materials and Manufacturing Engineering, University of Nottingham Ningbo China, Ningbo 315100, China; State Key Laboratory of Quantitative Synthetic Biology, Shenzhen Institute of Synthetic Biology, Shenzhen Institutes of Advanced Technology, Chinese Academy of Sciences, Shenzhen, Guangdong 518055, China

**Keywords:** host–microbe interaction, colonization resistance, probiotics, gut-on-chip

## Abstract

Intestinal microbiota resists pathogenic bacterial infections through colonization resistance, largely attributed to direct microbial competition. However, whether commensals can provide colonization resistance by remodeling the host epithelial niche remains underexplored. Here, by combining *in vivo* mouse models and a gut-on-chip model, this study demonstrates that the resistance of commensal *Escherichia coli* Nissle 1917 (EcN) against *Salmonella* Typhimurium (STm) infection is strictly dependent on EcN precolonization. Mechanistically, the EcN outer membrane protein MipA was identified as a key factor that induces the upregulation of integrin-linked kinase (ILK), thereby reinforcing tight junction integrity and restricting *Salmonella* infection. Engineering a nonprotective strain to express MipA was sufficient to confer resistance against *Salmonella* infection. This work reveals an epithelial defense mechanism triggered by a specific probiotic protein, with implications for developing preventive strategies against enteric infections.

## Graphical Abstract

**Graphical Abstract:**
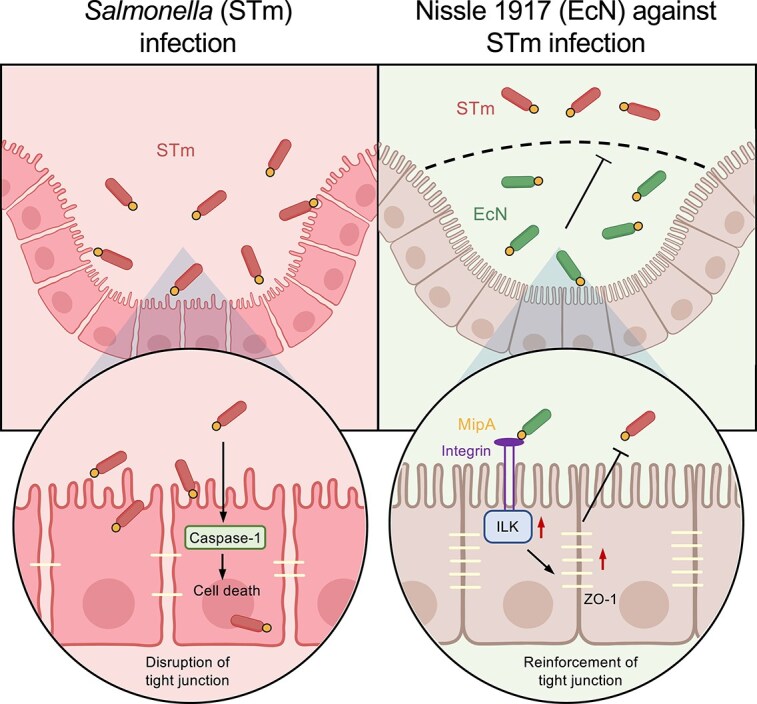

## Introduction

The integrity of the gastrointestinal tract is continuously challenged by enteric pathogens [1, 2], yet its stability is largely secured by colonization resistance, a fundamental ecological mechanism mediated by the resident microbiota [3–6]. The breakdown of colonization resistance is closely associated with dysbiosis and remains a significant driver of global health burden due to infectious diseases [7–9]. Therefore, understanding the molecular and ecological basis by which commensal bacteria establish and maintain colonization resistance represents a core question in microbial ecology [10].

Among the commensal microbiota, certain resident species are recognized for their probiotic function. A prime example is *Escherichia coli* Nissle 1917 (EcN) [11], a commensal strain specifically utilized as a probiotic for its ability to modulate host immunity, maintain intestinal homeostasis, and enhance resistance against pathogenic bacteria [12–14]. The protective efficacy of EcN is mainly attributed to direct microbial competition mechanisms. Once EcN stably adheres to the intestinal mucosa, EcN can directly inhibit the growth of pathogens by secreting bacteriocins [15, 16]. Recent studies have shown that EcN limits *Salmonella* colonization by effectively competing for essential but limiting nutrients, such as iron and nitrate, in the inflamed gut [17–19].

Beyond direct antagonism with pathogens, can established microbiota species remodel the host epithelial niche to enhance physical defense, thereby establishing a niche construction that is unfavorable to invaders? Although the theory of space occupation suggests that commensals physically block pathogen binding sites, physical exclusion alone may not fully account for colonization resistance [20]. Pathogens have evolved mechanisms to breach epithelial integrity [21–23]. Therefore, robust resistance often requires commensals to actively engage host pathways to promote intestinal barrier. Growing evidence suggests that intestinal microbiota engage broader, host-mediated defense systems [24, 25]. For instance, EcN administration has been shown to mitigate the epithelial barrier damage caused by enteropathogenic *E. coli* via inhibiting PKCζ activity [26] and to restore mucosal permeability in dextran sulfate sodium (DSS)-induced colitis models [27]. However, existing findings show that EcN restores epithelial barrier integrity, but it is underexplored how EcN proactively engages host molecular pathways to mobilize an active defense prior to infection.

**Figure 1:**
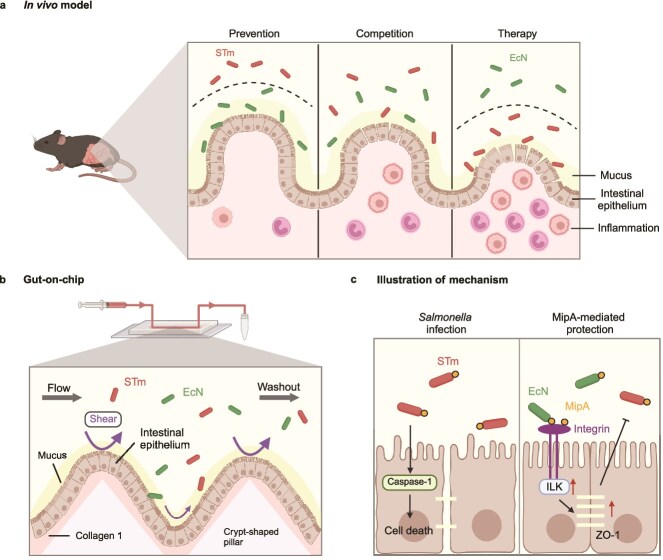
Schematic overview of the study approach. (a) The mouse model to evaluate the effects of three probiotic administration strategies: prevention, competition, and therapy. (b) The configuration of the gut-on-chip features a crypt-like scaffold for host–microbe coculture. Microfluidic flow provides nutrient and removes unattached bacterial cells from the microchannel. The flow also exerts shear stress on the crypt-like epithelial structure, with low shear stress in the crypt and high shear stress at the villus tips. (c) MipA-mediated prevention enhances the gut barrier against STm infection by promoting ILK and tight junction protein expression.

Here, this study investigates the underlying molecular basis by which the probiotic EcN utilizes host-regulatory pathways to establish resistance against *Salmonella* Typhimurium (STm) infection. To ensure physiological relevance and mechanistic precision, an integrative experimental design combining an *in vivo* mouse model with a 3D gut-on-chip was employed. The microfluidic platform incorporates computational fluid dynamics (CFD) simulations to accurately reproduce physiological fluid shear stress, creating a controlled micro-ecosystem for quantitative analysis (Fig. 1a and b). By leveraging this multiscale approach, the protection was found to depend on precolonization of EcN, and the outer membrane protein MipA was identified as the key effector. At the molecular level, MipA enhances the host barrier function by upregulating the expression of integrin-linked kinase (ILK) in epithelial cells, thereby limiting STm infection (Fig. 1c). These findings provide precise molecular evidence for host-mediated niche construction, expanding the understanding of colonization resistance and laying the foundation for the development of anti-infection strategies.

## Materials and methods

### Ethics approval and consent to participate

All animal experiments were approved by the Ethics Committee of Shenzhen Institute of Advanced Technology and performed in accordance with the guidelines of the Institutional Animal Care and Use Committee of Shenzhen Institutes of Advanced Technology (SIAT-IACUC-20230817-HCS-JHZX-HSQ-A2296-01).

### Cell lines

Caco-2 cell (cat# 1102HUM-NIFDC00087) and HT-29 cell (cat# 3101HUMSCSP5032) were supplied by the Cell Resource Center of Peking Union Medical College (Beijing, China). Cells were grown in Dulbecco’s Modified Eagle Medium (DMEM, Hyclone) containing 10% (*v/v*) heat-inactivated FBS (Gibco) and antibiotics (100 μg/ml penicillin and 100 μg/ml streptomycin, Hyclone). All the cells were cultured at 37°C in a 5% CO_2_ atmosphere.

### Bacterial strains

Strain STm, EcN, and the *mipA* mutant derivative of the wild-type strain EcN, STm Δ*prgK,* and STm Δ*prgK*Δ*mipA* were used in this study. All strains were routinely grown aerobically in Luria–Bertani (LB) broth or on LB agar plates at 37°C. STm strain expressing the green fluorescent protein (GFP) and EcN expressing the red fluorescent protein (RFP) were grown in LB medium containing kanamycin (50 μg/ml) at 37°C.

### Mouse models

Male C57BL/6 mice (6 to 8 weeks old) were purchased from the Military Medical Science Academy Laboratory (Beijing, China). Animals were housed under a controlled temperature (22–24°C), stable humidity (40%–60%), and 12-h light/dark cycle with ad libitum access to food and water.

C57BL/6 mice were treated with streptomycin [28] and kanamycin (100 μl of a 200 mg per ml solution in sterile water) 1 day before bacterial gavage. For the single-strain colonization experiment, mice were gavaged the next day with 0.1 ml sterile water containing 1 × 10^9^ CFU of either STm or EcN. Intestinal and liver tissues were collected on Days 1, 3, 5, and 7 postadministration, and hematoxylin and eosin (H&E) staining was performed to observe the different effects of STm and EcN on mice, respectively.

To evaluate the efficacy of EcN, mice were divided into three experimental groups (prevention, competition, therapy) based on the intervention strategy. For prevention, mice were orally administered EcN (1 × 10^9^ CFU per mouse) 1 day after antibiotic treatment. After a colonization period of 7 days, the mice were challenged with STm (1 × 10^9^ CFU per mouse). For competition, mice were orally inoculated with a 1:1 mixture of 0.1 ml sterile water containing 1 × 10^9^ CFU each of STm and EcN, 1 day after antibiotic treatment. For therapy, mice were first infected with STm (1 × 10^9^ CFU per mouse) 1 day after antibiotics. Two-day postinfection, mice were treated with EcN (1 × 10^9^ CFU per mouse). Mice were euthanized on Day 7, and intestinal tissues were collected, fixed in 10% formalin for H&E staining and immunostaining.

To quantify the bacterial colonization, fecal samples were collected each day, and intestinal tissue samples were collected on Day 7. The collected samples were weighed and homogenized in 1 ml of sterile phosphate-buffered saline (PBS). The bacterial load for STm and EcN strains was then determined by plating serial tenfold dilutions on kanamycin-resistant agar plates.

### Histopathology analysis

Tissue samples from mice were sectioned and stained with H&E. Pathological scoring of the intestinal tissues was performed in a blinded manner according to the modified published method [29]. Each sample was assessed for the severity of inflammation (score 0–3) and epithelial damage (score 0–3). The individual scores for these parameters were summed, with the total score interpreted as follows: 0–1 (normal), 2–4 (mild pathology), and 5–6 (severe pathology).

### Fabrication of the 3D gut-on-chip

The microfluidic chip device was constructed with a polydimethylsiloxane (PDMS; Sylgard, Dow Corning) microchannel layer, a glass coverslip, and a crypt-shaped scaffold using the conventional soft lithography procedures [30]. Briefly, a prepolymer base and curing agent for PDMS were mixed at a 10:1 (*w/w*) ratio, poured onto a master mold featuring a photoresist (SU8 2100) pattern, and cured at 80°C for 1 h. The microchannel measured 1.5 mm wide, 10 mm long, and 0.2 mm high. The crypt-shaped scaffold was produced using a patterned silicon substrate as previously described [31]. A PDMS mixture (prepolymer base to curing agent ratio of 15:1, *w/w*) was applied to the silicon template by spin-coating and cured (80°C, 1 h) to obtain the micropyramidal scaffold. The scaffold and a glass slide were exposed to plasma (Harrick Plasma, USA) for preliminary bonding. The final assembly was achieved by sealing the microchannel layer onto the sealed glass slide [32].

Prior to cell culture, the microchannel was sterilized by 75% ethanol, followed by exposure UV-ozone for 30 min. Type I collagen (Gibco, 100 μg/ml) was injected into the microchannel for surface coating. Intestinal cells (Caco-2 and HT-29) were dissociated, and resuspended in DMEM to a final density of 1 × 10^7^ cells per ml. The mixture of cell suspension (Caco-2: HT-29 = 3:1) was loaded into the microchannel and incubated for 2 h at 37°C with 5% CO₂ without perfusion to facilitate attachment. HT-29 is a mucin-secreting cell line, and the cultivation of these two types of cells can compensate for the lack of mucus production in Caco-2 monocultures. After cell settlement, the culture medium was perfused to the microchannel at 40 μl/h for 3 days to obtain gut-on-chip for further experiments.

### Cocultures of intestinal cells and bacteria in the gut-on-chip

Overnight cultures of EcN, STm were grown in LB medium at 37°C, with shaking at 225 rpm. The following day EcN and STm were diluted into the fresh medium till the exponential phase and suspended in DMEM medium. For host–microbe coculture, the gut-on-chip device had been preconditioned in the antibiotic-free culture medium for 12 h before microbial cells were introduced. After attachment of microbial cells for 30 min without perfusion, the flow of culture medium was set at 40 μl/h (details please refer to the Supplementary Information). To quantify the colonized bacteria on the epithelium, the chip was rinsed with PBS to remove nonadherent bacteria. Trypsin was then introduced into the microchannels to digest the intestinal cells. The cell suspension was serially diluted and plated onto agar plates to determine the bacterial counts. To visualize the bacterial colonization, fluorescent images were captured using a confocal microscope. The fluorescence intensity was quantified with ImageJ to evaluate bacterial density.

### Immunofluorescence staining analysis

Intestinal epithelial cells in the gut-on-chip were washed with PBS, fixed with 4% paraformaldehyde for 15 min, and washed with additional PBS subsequently. Cells were then permeabilized with 0.25% Triton X-100 in PBS for 10 min, followed by incubation in 5% bovine serum albumin in PBS for 1 h. After incubating with the primary antibody overnight, the corresponding secondary antibody was added and incubated for 1 h, followed by washing with PBS (see Supplementary Information for more details). The nuclei were stained with DAPI (KGA215-10, Keygen) before fluorescence imaging. To quantify the expression levels of ZO-1, ILK, and cleaved caspase-1, ImageJ was used to measure the fluorescence density.

### Proteomics analysis

To characterize the epithelial responses in the gut-on-chip model, protein extraction was performed directly in the microfluidic channel. Samples were then processed for liquid chromatography–tandem mass spectrometry (LC–MS/MS) analysis following an established workflow (detailed in Supplementary Information). The MS/MS datasets were searched against the Swiss-Prot database [*Homo sapiens* (20191227), STm (20191227), and EcN (20191227)] downloaded from UniProt with MaxQuant 1.6.10.43. The search parameters included precursor and fragment mass tolerances of 20 ppm and 0.5 Da, respectively. The basic data processing refers to the published method [33]. The DEP R package based on limma method was used for determining differentially expressed human proteins in the gut-on-chip model. Proteins with |log_2_ (fold change)| ≥ 1 and *P*-value ≤ .05 were defined as being differentially expressed.

### Immunoblots

Cells from the gut-on-chip were lysed in SDS lysis buffer. The lysed cells were then centrifuged at 20 000 g for 10 min at 4°C, and the amount of protein in each supernatant was measured by the BCA reagent (Thermo Scientific, 23227). Protein samples were resolved on 10% SDS-PAGE gels and subsequently transferred onto nitrocellulose membranes. The membranes were blocked with 1% nonfat dry milk in TBST for 1 h at room temperature, followed by an overnight incubation at 4°C with the primary antibody against ILK (ab150077, Abcam) diluted in TBST. After washing, the membranes were exposed to the secondary antibody (ab288151, Abcam) in TBST for 1 h at room temperature. The protein bands were captured with a ChemiDoc MP Imaging System (Bio-Rad) and quantified using ImageJ.

### Integrin-linked kinase inhibition assay

Caco-2 cells (5 × 10^5^) were seeded into each well of a 6-well plate and incubated overnight in a 37°C incubator with 5% CO_2_. The next day, the epithelial monolayers were treated with the antagonist (2 μM OSU-T315) of ILK for 4 h. To assess the impact of the ILK inhibitor on the epithelial barrier, immunofluorescence staining for ZO-1 protein was performed. ILK antagonist-pretreated epithelial cells were infected with STm at a multiplicity of infection (MOI) of 10 for 4 h to ensure bacterial invasion without immediate cytotoxicity. Following infection, a gentamicin protection assay was employed to quantify intracellular bacteria [34]. The epithelial cells were incubated in DMEM supplemented with 1% FBS and 100 μg/ml gentamicin for 15 min to eliminate extracellular bacteria. After washing with PBS, the cells were lysed using DMEM containing 0.1% Triton X-100. The lysates were then serially diluted, plated onto agar plates, and incubated overnight at 37°C.

### Construction of *Escherichia coli* Nissle 1917 and *Salmonella* Typhimurium mutant strain

The *mipA* gene (located at 1955272–1956018) was deleted from the chromosomes of both EcN (GenBank accession no. CP_007799.1) and STm strain 14028 *ΔprgK* (provided by Prof. Nan Li) using the lambda Red recombination system with plasmid pSIM6 [35].

### Statistics analysis

For statistical analyses, differences were calculated using a student’s *t*-test to evaluate the significant difference between every two groups by GraphPad Prism 9 (GraphPad Software Inc.). Differences between groups were considered statistically significant when *P* < .05 and are indicated with asterisks: ^*^P < .05, ^**^P < .01, and ^***^P < .001.

## Results

### Oral administration of probiotics prevents *Salmonella* Typhimurium infection in mice

**Figure 2:**
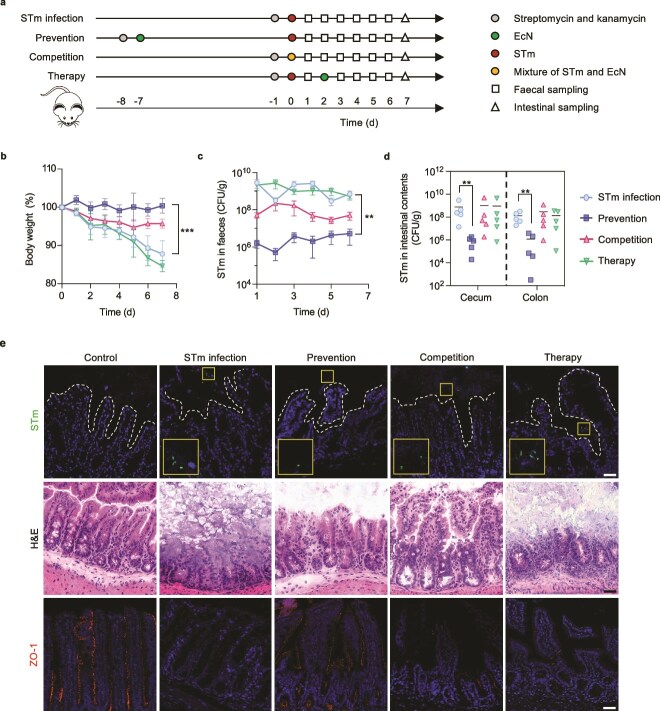
Probiotic EcN prevents STm colonization and intestinal barrier loss in a mouse model. (a) Experimental scheme for examining effects of three EcN intervention strategies on the mice model against STm infection. (b) Comparison of body weight over time. (c) Comparison of STm loads in feces samples. (d) Amounts of STm in the cecum and colon contents on Day 7 after infection. (e) Representative micrographs of cecum tissue sections. Top: fluorescence images showing the colonization of STm; middle: H&E-stained images; bottom: immunofluorescence images stained for tight junction protein ZO-1. In the top panel, the white dotted lines represent the boundary of epithelium, and the bottom left insets are the enlarged images of the boxed areas. Scale bar, 25 μm. Data (b, c) are presented as mean ± SD (*n* = 5). Significance was calculated by unpaired *t*-test. ^**^P < .01, and ^***^P < .001.

To investigate the protective effects of EcN, three intervention strategies were designed as prevention, competition, and therapy, corresponding to the administration of EcN before, during, and after pathogenic *Salmonella* infection, respectively (Fig. 2a). In the absence of EcN, mice infected with STm developed diarrhea and hematochezia within 1 week, exhibited slightly decreased activity, and experienced a body weight loss of 12% by Day 7 relative to initial weight (*P* < .001) (Fig. 2b). As the infection progressed, H&E-stained tissue sections showed that STm gradually destroyed the epithelial tissue structure, resulting in the loss of crypt structure, depletion of goblet cells, and infiltration of immune cells (Fig. S1). When mice were loaded with EcN alone, there was no inflammatory response (Figs S2 and S3a). The mice in the therapy group (EcN post-treated) developed diarrhea, and weight loss was statistically indistinguishable from the STm-infected group (*P* = .760), confirming that therapeutic intervention failed to mitigate the disease. The prevention group (EcN precolonized) displayed a significant reduction in weight loss compared to the STm-infected group (*P* < .001). The competition treatment, where mice were challenged with EcN and STm simultaneously, conferred partial protective effects, though these effects were much diminished compared to the prevention group (*P* = .002) (Fig. 2b).

The impact of EcN oral treatment on STm colonization was also assessed in the intestine. In the prevention group, the STm level in feces was sharply reduced to 10^6^ CFU/g. The therapy group had a similar level to the STm-infected group at 10^9^ CFU/g (*P* = .937), whereas the competition group stayed around 10^8^ CFU/g (Fig. 2c). Consistent with these observations, the burdens of STm adhered to the intestine, particularly in the cecum and colon, were significantly lower (~100-fold reduction) in the prevention group on Day 7 postinfection (Fig. 2d and e), whereas the number of EcN remained around 10^8^ CFU/g (Fig. S3b). Decreasing the precolonization time of EcN by 2 days still prevented STm colonization (Fig. S3d and e).

Histology sections from the therapy group showed complete loss of epithelial structure, flattened villi, and damaged submucosa, which reflected the severe inflammatory effects of STm infection. The competition group had relatively more crypt structures but still exhibited a partial absence of mucosal epithelium. In contrast, an intact mucosal structure was retained in the prevention group, almost identical to the noninfected control group (Fig. 2e and Fig. S3c). Furthermore, immunofluorescence characterization of the intestinal epithelial tight junction protein ZO-1 revealed that the fluorescence intensity of ZO-1 in the prevention group was comparable to that of the control group, which was much higher than that in the competition or therapy groups (Fig. 2e). These results indicated that the precolonization of EcN could greatly limit STm pathogenesis and protect the tissue integrity of intestinal epithelium.

### Establishing the human gut *Salmonella* infection model in vitro

**Figure 3:**
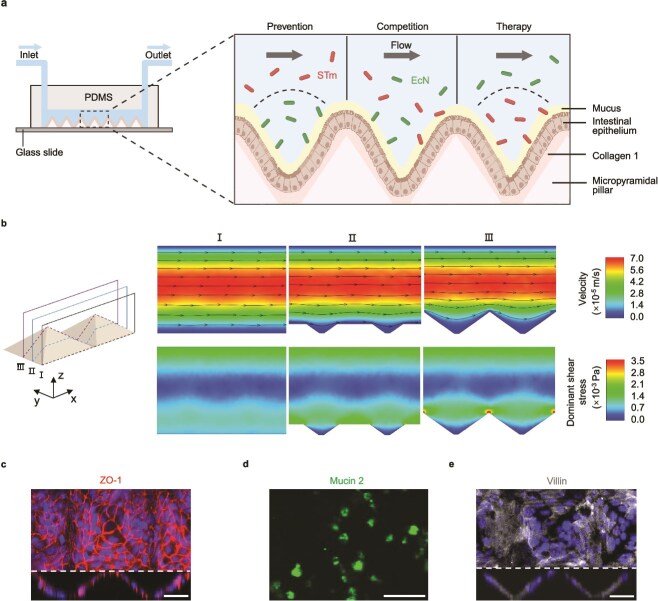
Characterization of the microfluidic human gut-on-chip model. (a) Schematic of the microfluidic human gut-on-chip. (b) Cross-sectional visualization of the streamwise velocity (the velocity component in the x direction, right top) and the shear stress *τ*_zx_ (i.e. the stress in the x direction on the surface normal to the z-axis, right bottom) in the xz planes (left) at *y* = 0.755 mm (I), *y* = 0.790 mm (II), and y = 0.825 mm (III). Solid black lines with arrows are the streamlines of the flow. (c) Confocal z-stack immunofluorescence image showing epithelial tight junction (ZO-1) within the human gut-on-chip (top panel), and cross-section image (on the xz plane) denoting the created crypt-villi microstructure (bottom panel). Scale bar, 50 μm. (d) Micrograph displaying the expression of mucus protein (Mucin 2) in the human gut-on-chip. Scale bar, 100 μm. (e) Confocal z-stack immunofluorescence image stained for intestinal differentiation marker Villin within the human gut-on-chip (top panel), and cross-section image (on the xz plane) denoting the created crypt-villi microstructure (bottom panel). Scale bar, 50 μm.

To explore the contributions of EcN to block intestinal pathogen infection and unveil the crosstalk between host cells and gut microbiota, a microfluidic device for a human gut-on-chip model was developed to simulate the intestinal microenvironments. The device differs from conventional intestinal organ chips in that its main channel was fabricated with a crypt-shaped scaffold (consisting of an array of micropyramidal pillars) instead of a planar substrate [36] at the channel bottom (Fig. 3a). Human intestinal epithelium (enterocyte-like Caco-2 cells and goblet-like HT-29 cells) was then cocultured on this 3D crypt-shaped scaffold to form the intestinal epithelial layer. The distinct structural design not only promoted epithelial growth in microstructures that more resemble crypt-villi but also provided a dynamic cell culture environment closer to realistic physiological conditions, thereby improving the biological relevance and functionality of this model.

It has been shown that the flow of digestive fluids in the human gut exposes intestinal epithelial cells to their viscous forces, which as a key mechanical stimulus, promotes epithelial differentiation [37], brush border formation [38], and the maturation of tight junctions [39]. In this study, a 3D CFD simulation was performed (for details see Supplemental information) to reveal the fields of both velocity and viscous stress in the presence of the crypt-shaped scaffold (Fig. 3b) and provide a specific analysis of the flow effects on bacterial transport and relevant biological characteristics. The culture medium was driven through the main channel at a volumetric flow rate of 40 μl/h [40], corresponding to a very low Reynolds number, *Re* = 1.3 × 10^−2^. Under this condition, the flow in the channel was laminar and viscously dominant. The streamlines far away from the channel bottom (i.e. in the bulk flow region) were straight and parallel, whereas those close to micropyramids were significantly distorted toward the lateral faces of pyramids (Fig. 3b). The distorted streams directly modulated host–microbe interactions-the streams along them carried bacteria away from the bulk flow region and into the cavity between adjacent micropyramids (see III, Fig. 3b), facilitating cell colonization within crypt-like niches. It was also found that the shear stress *τ*_zx_ (Fig. S4b–d) was predominant in most channel regions, with magnitudes on the walls ranging from 0.0005 to 0.003 Pa, which were well-agreed with the range of realistic wall shear stress in the intestine [40, 41]. As for the other two stresses, *τ*_xx_ and *τ*_yx_, they exhibited non-negligible values in the vicinity of micropyramids (Fig. S4c and d). In particular, at the apex and along four lateral edges of micropyramids, *τ*_zx_, *τ*_xx_ and *τ*_yx_, have all increased to larger values (Fig. 3b and Fig. S4e). Such a complex force field caused bacteria near structural edges to be apt to tumble and follow circular trajectories [42], and as a consequence, they experienced longer contact time with the pyramid surfaces, which promoted their adhesion [43]. By CFD simulation, our numerical results demonstrate that, with the compound effects of shear (off-diagonal components) and normal (diagonal components) stresses exerted by the ambient flowing fluid, cell behaviors in a real gut extend far beyond a 2D description, and are subject to fundamentally more complicated near-wall dynamic mechanisms compared to those in conventional planar-channel chips.

**Figure 4:**
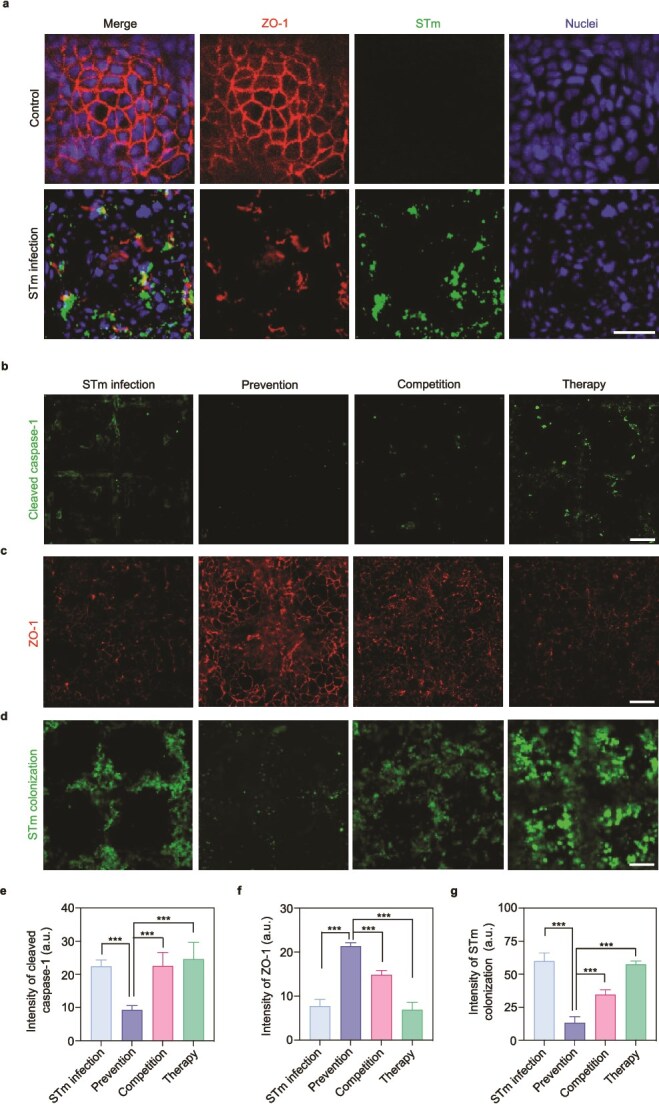
EcN prevents STm infection in the human gut-on-chip. (a) Immunofluorescence micrographs showing the distribution of tight junction protein ZO-1 within the gut-on-chip in the absence (control) and presence of STm infection for 12 h. (b) Immunofluorescence micrographs of intestinal cells stained for cleaved caspase-1. (c) Integrity of tight junction protein ZO-1 within the gut-on-chip in each experimental group. (d) Fluorescence images of STm colonization in the gut-on-chip. (e–g) Quantification of the averaged fluorescence intensity of cleaved caspase-1 (e), ZO-1 (f), and colonized STm (g). Scale bar, 50 μm. Data (e–g) are presented as mean ± SD (*n* = 10). Significance was calculated by unpaired *t*-test. ^***^P < .001.

To confirm that our gut-on-chip recapitulates critical epithelial features, the distribution and expression of key functional markers were analyzed. Immunofluorescence staining of ZO-1 revealed clear, continuous networks at cell–cell boundaries, suggesting the establishment of epithelial barrier. To assess mucus production, the chip was stained for Mucin 2, the predominant gel-forming mucin in the gut. Clear Mucin 2-positive signals were detected over the epithelial surface (Fig. 3d), indicating that our gut-on-chip possesses the physiological capability to secrete mucus, consistent with established gut-on-chip models [36, 44]. In addition, the intestinal epithelium in the chip strongly expressed the brush border marker Villin. The expression level is comparable to that in native human intestinal tissue and established tissue-derived models [45, 46], confirming successful differentiation (Fig. 3e). Collectively, these results demonstrate that our system not only recapitulates villus-like structures but also maintains barrier integrity, secretes mucus, and achieves cellular differentiation in a physiologically relevant manner. To model the STm infection, GFP-labeled STm was introduced into the microchannel. Fluorescent data confirmed that STm infected the human intestinal epithelium and disrupted tight junctions as visualized by the immunofluorescence staining (Fig. 4a). STm proliferated to high abundance within 12 h (Fig. S5a and b). When EcN was introduced alone into the gut-on-chip, EcN also achieved stable proliferation (Fig. S5c) but did not destroy tight junctions. These results indicate that the gut-on-chip provides a physiologically relevant system to study bacterial infection.

### Pre-administration of *Escherichia coli* inhibits *Salmonella* Typhimurium infection in the human gut-on-chip

*Salmonella* invasion into intestinal epithelial cells can activate caspase-1 to induce cell pyroptosis [47]. The effects of three intervention strategies on the STm-induced intestinal changes were then analyzed in vitro, based on the crypt-like gut-on-chip. Immunofluorescence staining showed that EcN precolonization prevented activation of caspase-1 and relieved disruption of epithelial tight junctions (Fig. 4b, c, e, and f). The colonization of STm was strongly inhibited in the prevention groups compared to the other two strategies (*P* < .001) (Fig. 4d and g). Moreover, the treatment dosage and the precolonization time were optimized in the prevention strategy. Based on our results, a longer precaution of EcN prior to pathogenic infection led to less STm colonization (Fig. S5e). After 8 h of precolonization, the number of adhered STm significantly dropped to half of that observed after 2 h (*P* = .029). Additionally, we found that a comparable dosage of EcN (10^9^ CFU/ml) was required to provide a significantly preventive effect (*P* < .001). Otherwise, if the concentration of EcN was reduced to only 1% of STm concentration (10^7^ CFU/ml), the prevention did not occur even with 8 h of EcN pretreatment (Fig. S5d and f). A higher dosage and longer precolonization time of probiotics resulted in a better effect in limiting STm colonization during the same treatment period. Together, these results reveal that the premedication of EcN is an effective strategy for disturbing STm infection, which is highly consistent with the *in vivo* results.

### Identification of integrin-linked kinase in epithelial cells as a regulator of the preventive effect

**Figure 5:**
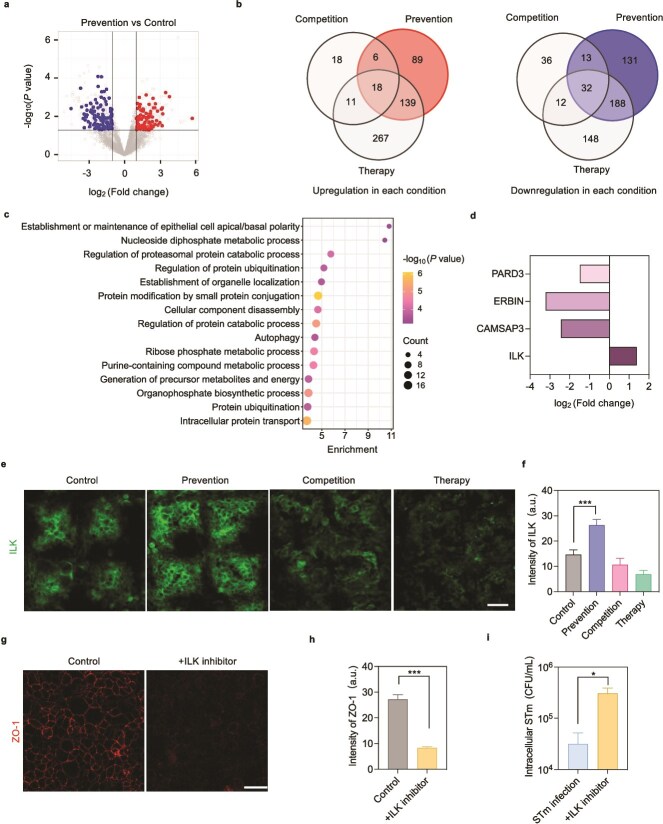
Quantitative proteomics reveals that ILK in epithelial cells mediates preventive effect. (a) Volcano plots of the prevention group versus the control group indicating significantly regulated proteins in prevention. Data points in the upper right section indicate proteins upregulated in the prevention group but not in the competition or therapy groups (fold change ≥2 and *P*-value ≤ .05); data points in the upper left section indicate proteins downregulated in the prevention group but not in the competition or therapy groups (0 ≤ fold change ≤ 0.5 and *P-*value ≤ .05). (b) Venn diagram comparing the proteins of intestinal cells under the prevention, competition, and therapy strategies (fold change ≥2 or 0 ≤ fold change ≤ 0.5, *P*-value ≤ .05). (c) Top 15 enriched GO terms (biological process) for differentially expressed proteins in the prevention group. (d) Expression levels of the differential proteins belonging to the establishment or maintenance of epithelial cell apical/basal polarity. (e) Comparison of ILK expression among the control, prevention, competition and therapy groups in the gut-on-chip. Scale bar, 50 μm. (f) Quantification of ILK fluorescence intensity in (e) (*n* = 10). (g) Immunofluorescence images of intestinal cells stained for ZO-1 in the presence of ILK inhibitor OSU-T315. Scale bar, 50 μm. (h) Quantification of ZO-1 fluorescence intensity in the presence of OSU-T315 (*n* = 10). (i) Quantification of intracellular STm in the presence of OSU-T315 (*n* = 3). Data (f, h, i) are presented as mean ± SD. Significance was calculated by unpaired *t*-test. ^*^P < .05 and ^***^P < .001; ns, not significant.

To understand how precolonization with EcN confers beneficial effects on the intestinal barrier, quantitative proteomics of epithelial cells from the prevention, competition, and therapy groups was performed. Compared with the control (bacteria-free) group, the prevention group showed differential expression of 616 proteins (252 increased, 364 decreased) (Fig. 5a). When comparing the differential expression profiles among the prevention, competition, and therapy groups, 220 proteins specifically expressed were detected in the prevention group (89 increased, 131 decreased) (Fig. 5b). Gene ontology (GO) analysis revealed that the biological process associated with the establishment or maintenance of epithelial cell apical/basal polarity was ranked top according to the enrichment score (Fig. 5c). Apical/basal polarity is critical for the formation and function of epithelial cells, as it determines both the positioning of adhesion molecules that tie cells together and adhesion junctions that act as a barrier to prevent paracellular diffusion [48, 49]. Several reported pathogens can perturb the epithelial barrier, causing a loss of apical/basal polarity [50]. By examining regulated proteins related to the establishment of epithelial cell apical/basal polarity, it was found that ILK was the only upregulated protein that might facilitate the preventive effect (Fig. 5d). ILK is an intracellular protein kinase and functions as a molecular actor to mediate the signaling pathways involved in multiple cellular processes [51]. Secreted bacterial effector proteins were also shown to be mediated by ILK to alter the stability of host cell attachment, suggesting a vital transduction role of ILK in bacteria–cell interactions [52–54]. Immunofluorescence and western blot analysis confirmed that the expression of ILK did increase in the prevention group, consistent with the proteomic analysis (Fig. 5e and f and Fig. S6a and b). Furthermore, treatment with an ILK inhibitor OSU-T315 [55] decreased ZO-1 expression and promoted STm invasion into intestinal epithelial cells (Fig. 5g–i and Fig. S6c and d). These results indicate that ILK is a critical factor mediating the production of tight junction proteins and restricting STm invasion of the epithelium in the presence of EcN colonization.

### MipA of *Escherichia coli* enhances integrin-linked kinase expression and intestinal epithelial tight junction

**Figure 6:**
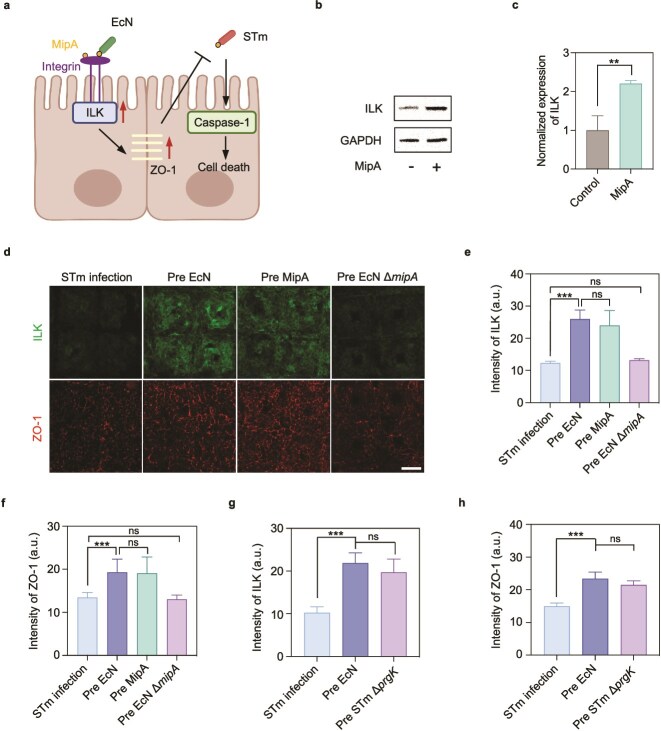
MipA of EcN activates ILK to maintain epithelial cell–cell junctional integrity. (a) Schematic diagram illustrating the mechanism that MipA induces the higher expression of ILK mediating preventive effects of EcN against STm infection. (b) Immunoblotting and (c) corresponding quantification of expressed ILK in intestinal cells when incubated with the purified MipA protein (10 μg/ml) for 4 h (*n* = 3). (d) Immunofluorescence micrographs of intestinal cells stained for ILK and ZO-1 in the gut-on-chip pretreated with MipA protein, EcN, or EcN mutant for 4 h prior to 4-h STm infection. Scale bar, 50 μm. (e, f) Quantification of the fluorescence intensity of ILK and ZO-1 as the conditions in (d) (*n* = 10). (g, h) Quantification of the fluorescence intensity of ILK and ZO-1 in cells pretreated with EcN or noninvasive STm (*n* = 10). Data (c, e–h) are presented as mean ± SD. Significance was calculated by unpaired *t*-test. ^**^P < .01 and ^***^P < .001; ns, not significant.

The bacterial outer membrane protein MipA has been reported to act as an adhesin that binds to α1β1 integrins to promote bacterial adhesion to cell surfaces [56]. ILK is an essential partner of the β1 integrin cytoplasmic tail to regulate diverse cellular processes [57]. As MipA also exists in EcN, we hypothesized that MipA of EcN binding to integrin β1 might regulate downstream ILK, and then mediate host cell response when EcN was precolonized (Fig. 6a). As a validation, purified MipA protein was incubated with intestinal cells (Fig. S7a). Western blot analysis showed that the ILK expression increased approximately 2.2-fold (*P* = .002) (Fig. 6b and c). Immunofluorescence staining further confirmed that both pure MipA protein and EcN increased the expression levels of ILK and ZO-1 as well (Fig. S7b–d). The pretreatment of MipA protein alone could also reduce the STm invasion into intestinal epithelial cells (Fig. S7e–g). To obtain more evidence, intestinal cells were then pretreated with the purified MipA, EcN, and EcN Δ*mipA* mutant, followed by STm infection. Both MipA and EcN, but not EcN Δ*mipA*, significantly induced the ILK expression (*P* < .001) and protected the tight junction of the intestinal epithelium (*P* < .001) compared to the STm infection group (Fig. 6d–f). Although the *mipA* gene in STm is highly homologous to that of EcN (identity >80%), STm did not exhibit MipA-mediated beneficial effects on cells. To explain this phenomenon, inner-membrane component PrgK responsible for STm type III secretion system (T3SS) [58] was knocked out, and it was observed that coculture with the noninvasive strain (STm Δ*prgK*) resulted in similar ILK (*P* = .123) and ZO-1 (*P* = .076) expression levels in intestinal cells to that of EcN groups (Fig. 6g and h and Fig. S8a). However, when MipA was knocked out (STm Δ*prgK*Δ*mipA*), the protective effect decreased, as reflected by the reduced expression of ZO-1 protein (Fig. S8b and c). Our results suggest that MipA should be a key factor that increases the ILK expression and improves the epithelial tight junction.

## Discussion

### MipA-mediated fortification of the epithelial niche

The intestinal barrier is a dynamic interface shaped by continuous interactions with commensal bacteria. This study demonstrates that the probiotic EcN can fortify this epithelial niche to prevent early invasion and tissue damage caused by *Salmonella*. Integrated *in vivo* and gut-on-chip micro-ecosystem models reveal that effective protection depends on EcN precolonization, implicating a host-mediated mechanism that maintains epithelial structure and tight junction integrity. Mechanistically, the EcN outer membrane protein MipA is identified as a host-protective factor that interacts with integrins to induce ILK expression, thereby reinforcing cell–cell junctions. Collectively, these findings depict a probiotic factor-driven strategy that modulates host responses to resist enteric STm infection.

### The defense mechanism of MipA-integrin-linked kinase regulation

A critical gap in understanding colonization resistance lies in deciphering how commensals communicate with the epithelium. Although the outer membrane protein MipA has been previously characterized primarily as an adhesin [56] and antibodies against the MipA protein can block the adhesion of enterotoxigenic *E. coli* [59, 60], the current study uncovers a distinct, signaling-dependent mode of action beyond simple physical attachment. Quantitative proteomic profiling provided direct molecular evidence that EcN precolonization specifically upregulates ILK expression in the host. Pharmacological inhibition of ILK was found to abrogate tight junction enhancement and increased STm invasion, establishing ILK as an essential node in the regulation of tight junction, which is consistent with previous report [61]. Based on these data and protein localization, it is proposed that MipA triggers an outside-in signaling cascade. Specifically, MipA interacts with β1 integrins, which likely recruits the assembly of focal adhesions, activates focal adhesion kinase (FAK) and the downstream PI3K/Akt pathway [56, 62]. This signaling cascade subsequently leads to the stabilization and transcriptional induction of ILK via NF-κB [63]. Thus, these findings redefine MipA not merely as a colonization factor for bacterial fitness, but as a bioactive effector that proactively engages host signaling to reinforce the epithelial barrier.

Although ILK is a known target for certain pathogen-host interactions, its modulation by probiotics presented here is a unique mechanism. Pathogens such as *Shigella flexneri* have been shown to hijack ILK signaling; *Shigella* effectors bind ILK to stabilize focal adhesions, preventing the detachment of infected cells to facilitate bacterial survival [53]. Distinct from this pathogenic hijacking, this study demonstrates that the probiotic outer membrane protein MipA actively upregulates ILK expression. Rather than merely stabilizing infected cells, this upregulation serves to reinforce the tight junction barrier to block STm invasion. Thus, these findings expand the understanding of ILK as a vital regulator of host defense and provide molecular evidence that upregulating ILK expression represents a viable strategy for preventing infection.

This host-mediated mechanism is distinguished from the classical view of colonization resistance. Previous studies predominantly emphasized direct microbial competition for limited nutrients [18, 19, 64] or space occupation [65, 66], where commensals occupy epithelial adhesion sites to physically block pathogen attachment. Although these exclusion mechanisms likely contribute to overall protection, the current data argue for a more complex, active mode of action. The observation that protection is strictly dependent on the specific expression of MipA rather than precolonization density suggests that occupation of adhesion sites is insufficient to explain the full extent of resistance. Instead, the MipA-ILK regulation represents an active remodeling of the epithelial niche, where EcN does not merely persist on the surface but actively instructs the epithelium to fortify its barrier. Furthermore, this pathway represents a paradigm shift from other reported barrier-enhancement mechanisms. For instance, EcN was previously shown to restore tight junctions by inhibiting EPEC-induced PKCζ activity [26]. Unlike such reactive strategies that function to repair cellular damage, MipA-mediated ILK upregulation functions as a proactive scaffold to stabilize cell adhesion and tight junction assembly prior to infection. Thus, though MipA is identified as necessary for the specific phenotype of ILK-mediated barrier enhancement, it likely functions synergistically with other EcN fitness factors to optimize host protection.

### Context-dependent function of MipA

MipA is highly conserved between EcN and STm, yet wild-type STm fails to confer barrier protection. This functional divergence is likely dictated by the signaling environment. Although STm employs a diverse arsenal of virulence factors—including the Type 1 and Type VI secretion systems (T1SS, T6SS) to facilitate colonization and persistence [67, 68], the T3SS serves as the dominant machinery for acute epithelial invasion. The potent invasive signals mainly triggered by the T3SS (such as SopE/SopB-mediated actin remodeling and junctional disruption) likely override the homeostatic stabilization signals provided by the MipA-ILK axis [69–71]. The observation that MipA confers protection only when the T3SS is disabled (STm Δ*prgK*) confirms that the protective potential of MipA is masked specifically by the pathogen's dominant invasive machinery. Collectively, these findings imply that the MipA-ILK axis represents a more generalized mechanism of symbiotic crosstalk. It proposes a broader paradigm where conserved bacterial adhesin can function as universal commensal cues to reinforce epithelial integrity.

### Technological innovation

The microfluidic gut-on-chip system was employed to recapitulate the critical microenvironment of the human intestine and to isolate specific intestinal cell–microbe interactions. A micropyramid array was fabricated to simulate the crypt-villus architecture, promoting epithelial monolayer formation through an optimized geometry that mitigates the gravitational limitations associated with vertical structures [72, 73]. Furthermore, CFD simulations revealed that, in addition to shear stress, the normal stress exerted by fluid flow significantly impacts the host–microbe interactions, which has never been pointed out in previous literature. The flexibility of precise flow control not only allowed us to mimic intestinal flow [40], replenish fresh medium, remove free bacteria and toxic metabolites [74], but enabled us to switch different microbial species for different intervention strategies to decipher the mechanisms. Outcomes from this system closely paralleled observations in mouse models, supporting its utility as a translational platform to investigate crosstalk between intestinal epithelium and microbiota.

### Clinical implications and limitations

Clinically, EcN exhibited strong robust prophylactic efficacy against pathogen infections. The protective effect was observed to be time- and dose-dependent in the gut-on-chip, aligning with effective dosages reported in colitis trials [75]. These findings suggest that a preventive strategy based on early EcN administration against STm infection is translationally feasible [76]. However, certain limitations are acknowledged. Even though the interaction between MipA and integrin β1 is supported by functional phenotypes and previous literature [52, 77], direct biochemical evidence of the MipA-integrin binding was not provided in this study. Future structural or binding analysis will be valuable to fully elucidate the precise molecular interaction.

In summary, this study establishes a crucial molecular framework for understanding how probiotic EcN resist against STm infection. By integrating *in vivo* animal models, gut-on-chip microecosystems, and quantitative proteomics, we identified an anti-infection mechanism driven by the commensal outer membrane protein MipA but mediated by the host. MipA fortifies the epithelial barrier by activating ILK expression, thereby enhancing niche defense against invasion. These insights not only advance the fundamental understanding of host–microbe ecological interplay but also highlight the MipA-ILK axis as a promising target against drug-resistant intestinal pathogens.

## Supplementary Material

wrag054_Supplemental_File

## Data Availability

All data supporting the findings of this study are available in the article and supplementary information. The mass spectrometry data have been deposited to the ProteomeXchange Consortium via the PRIDE partner repository with the dataset identifier PXD068469.
