# Supplementary material for: Cellular membrane protein MipA from E.coli Nissle 1917 protects against Salmonella infection
Source: ISME J. 2026 Mar 13;20(1):wrag054. doi: 10.1093/ismejo/wrag054 (PMC13077295; doi:10.1093/ismejo/wrag054)
Supplement: wrag054_Supplemental_File [file wrag054_supplemental_file.docx]

**Supplemental information**

# Cellular membrane protein MipA from *E.coli* Nissle 1917 protects against *Salmonella* infection

Yunqing Xiang^1,2^, Huipai peng^1^, Yanxia Li^3^, Hui Wen^1^, Nan Li^1^, Xiaozhi Liu^3^, Guodong Yuan^4^, Yong Shi^2*^, and Shuqiang Huang^1,5*^

^1^State Key Laboratory of Quantitative Synthetic Biology, Shenzhen Institute of Synthetic Biology, Shenzhen Institutes of Advanced Technology, Chinese Academy of Sciences, Shenzhen, Guangdong 518055, China

^2^Department of Mechanical, Materials and Manufacturing Engineering, University of Nottingham Ningbo China, Ningbo 315100, China

^3^Tianjin Key Laboratory of Epigenetics for Organ Development of Premature Infants, Tianjin Fifth Central Hospital, Tianjin 300450, China

^4^The State Key Laboratory of Superlattices and Microstructures, Institute of Semiconductors, Chinese Academy of Sciences, Beijing 100083, China

^5^Lead contact

*Corresponding authors: Yong Shi, Shuqiang Huang

Email: Yong.Shi@nottingham.edu.cn, shuqiang.huang@siat.ac.cn

**This file includes:**

Supplementary Methods

Figures S1 to S8

**Supplementary Methods**

**Mouse models for the** **short-term infection**

For short-term animal experiments, the duration of STm infection was shortened to 3 days. In the prevention experiment, on day 2 after EcN pre-treatment, mice were treated with 1×10^9^ CFU of STm for 3 days. Mice in the competition experiment were orally inoculated with a 1:1 mixture of STm and EcN resuspension for 3 days. In the therapy experiment, on day 1 after STm infection, mice were treated with 1×10^9^ CFU of EcN for 2 days.

**Bacterial-epithelial cells interaction**

To model STm infection, the cultured microfluidic chips were infected with STm (10^9^ CFU/mL) in DMEM containing 10% FBS at the MOI of 10 for 12 h with flow. To mimic prevention strategy, the EcN suspension (10^9^ CFU/mL) was introduced into the microchannel. After that, it flowed for 12 h, and the chips were infected with STm for the next 12 h. In the competition scenario, the mixture of STm and EcN (1:1) was loaded into the microchannel, and followed by 12-h co-culturing. As to the therapy experiments, STm was first loaded into the microchannel for 12 h flow culture, and then the EcN suspension was introduced for the next 12 h.

For all co-culture experiments in Fig. 6, the gut-on-chips were pre-treated with EcN, protein MipA, EcN mutant, and STm mutant, respectively, for 4 h, followed by STm infection for the next 4 h. For co-culture experiments (Fig. S8b,c), 5 × 10^5^ Caco-2 cells were grown per well in a 6-well culture plate in a 37°C incubator with a 5% CO_2_ overnight. The next day the epithelial monolayers were pre-treated with STm mutants for 4 h. Then, the epithelial cells were infected with STm at the MOI of 10 for the next 4 h.

For the quantification of bacterial colonization, at the end of the infection, the chip was rinsed with PBS to remove unadhered bacteria. The trypsin was then added to the microchannel to digest the cells. Finally, cell suspensions were serially diluted onto agar in an incubator at 37°C overnight.

**Numerical simulation**

To describe the steady-state flow in the gut-on-chip at a low Reynolds number, the 3D incompressible Navier-Stokes equations were numerically solved using COMSOL Multiphysics 6.0. The chip consisted of a straight channel with a length of 1 cm (x direction), a width of 1.5 mm (y direction), and a height of 200 μm (z direction), featuring a crypt-shaped scaffold at its bottom to accommodate the cells. Such a basal crypt structure was composed of an ordered array of micropyramids, each measuring 50 μm high and having a square base of 140 μm×140 μm. All properties were set the same values as those of water, and no-slip boundary condition was employed at the solid boundaries, including the channel walls and micropyramid surfaces. A volumetric flow rate of 40 μL/h was specified at the channel inlet, yielding a very low Reynolds number (defined by *Re* = *ρUD_H_/**μ*) of 1.3×10^-2^. *ρ* and *μ* represent the density and viscosity of the culture medium, respectively. *D_H_* and *U* refer to the hydraulic diameter and average streamwise velocity, computed by approximating the channel cross-section as rectangular. By simulation, the fields of velocity **u**(*u,v,w*) and viscous stress **τ** = *μ*[∇**u+(**∇**u)***^T^*] were obtained in the chip, where ( ⋅ )*^T^* denotes the transpose.

**Immunostaining assay**

For immunofluorescence staining analysis, after cells were washed and fixed, Alexa Fluor 594-conjugated mouse anti-human ZO-1 monoclonal antibody (339194, Thermo Fisher, 1:100 dilution), villin antibody (PA5-29078, Thermo Fisher, 1:100 dilution), anti-human integrin linked ILK antibody (ab74336, Abcam, 1:500 dilution), caspase-1 antibody (PA5-38099, Thermo Fisher, 1:100 dilution) and Alexa Fluor 488-conjugated monoclonal antibody against human Mucin 2 (sc-7314, Santa Cruz, 1:100 dilution) were added and incubated overnight at 4°C respectively, and washed with PBS again. For Villin, cleaved caspase-1 and ILK staining, secondary fluorescent antibodies (ab150077, Abcam, 1:1000 dilution) were added to incubate for 1 h and washed with PBS.

**Tissue preparation for mass spectrometry analysis**

To characterize the epithelial response in the gut-on-chip model, protein extraction was performed directly within the microfluidic channels. Briefly, after removing the culture medium, cells were lysed on-chip using SDT lysate (1% SDS, 100 mM Tris-Hcl pH 7.8, 1 mM DTT, 1 mM MgCl_2_, and protease inhibitor cocktail) and incubated at 37°C for 30 min. The lysates were collected, sonicated, and centrifuged at 20000 g for 10 min at 4°C. Protein concentration in the supernatant was determined using the BCA reagent (Thermo Scientific, 23227). Approximately 25 μg protein solution was reduced and has been alkylated for 30 min at 37°C using 1 μL 0.5 M TCEP, and 2 μL 1 M CAA. LysC digestion was carried out with 1 mg of LysC for 3 h at the room temperature. Tryptic digestion was performed overnight after adding 4 volumes of 50 mM ammonium bicarbonate buffer, then followed by 1 mg trypsin. The following day digestion was stopped by adding 1% TFA. Finally, peptides were desalted on C18 Stage Tips and stored at -80°C.

**Liquid chromatography (LC)-mass spectrometry (MS) analysis**

Peptides were reconstituted in 0.1% formic acid and separated on reversed-phase columns using an Ultimat 3000 RSLCnano System (Thermo Fisher Scientific) coupled to Orbitrap Q-Exactive HF (Thermo Fisher Scientific). A 120-minute gradient (buffer A: 0.1% FA in water, buffer B: 0.1% FA in 80% ACN) was applied at 300 nL/min. Peptides were ionized and analyzed on the Q-Exactive HF mass spectrometer operating in positive mode with a 2.1 kV spray voltage and 275°C transfer tube temperature. Full MS scans (m/z 350-2000) were recorded at a 60,000 resolution in the Orbitrap. HCD fragmentation was set to a 28% normalized collision energy. The primary MS2 parameters were an AGC target value of 5×10⁴, a maximum injection time of 50 ms, and a dynamic exclusion period of 45 s.

**Purification of MipA**

The outer membrane protein MipA expression plasmid was transferred into *E. coli* BL21 (DE3). Following overnight growth on LB agar plates with kanamycin (50 µg/mL) at 37°C, a single colony was isolated to inoculate a 2 mL LB starter culture containing kanamycin (50 mg/mL). Subsequently, for large-scale expression, a 1:100 dilution of the starter culture was prepared by adding 4 mL into 400 mL of fresh LB medium and shaken at 220 rpm at 37°C until the optical density at 600 nm reached approximately 0.4. Protein expression was induced with 0.5 mM IPTG at 16°C for 20 h. The bacterial cells were then harvested by centrifugation at 8000 g for 20 min at 4°C. The LB medium supernatant was discarded and the cells were resuspended with cold PBS. Bacteria were lysis by a high-pressure homogenizer. For those small-scale expression trials, the bacterial soluble protein was collected by the Qproteome Bacterial Protein Prep Kit (Qiagen, 37900).

For MipA protein purification, the Ni-NTA spin column was pre-equilibrated with the binding buffer containing 2 mM PB buffer, 500 mM NaCl, and 5 mM imidazole, pH 7. To purify the 6×His-tagged protein, large-scale IMAC was conducted using an AKTA Purification System (Cytiva) and a 5 mL HisTrap HP column. Elution was achieved with a buffer of 20 mM PB, 500 mM NaCl, and 500 mM imidazole at pH 7. The concentration of the purified protein was then measured by the Pierce BCA Protein Assay Kit (Thermo Scientific, 23227).

**
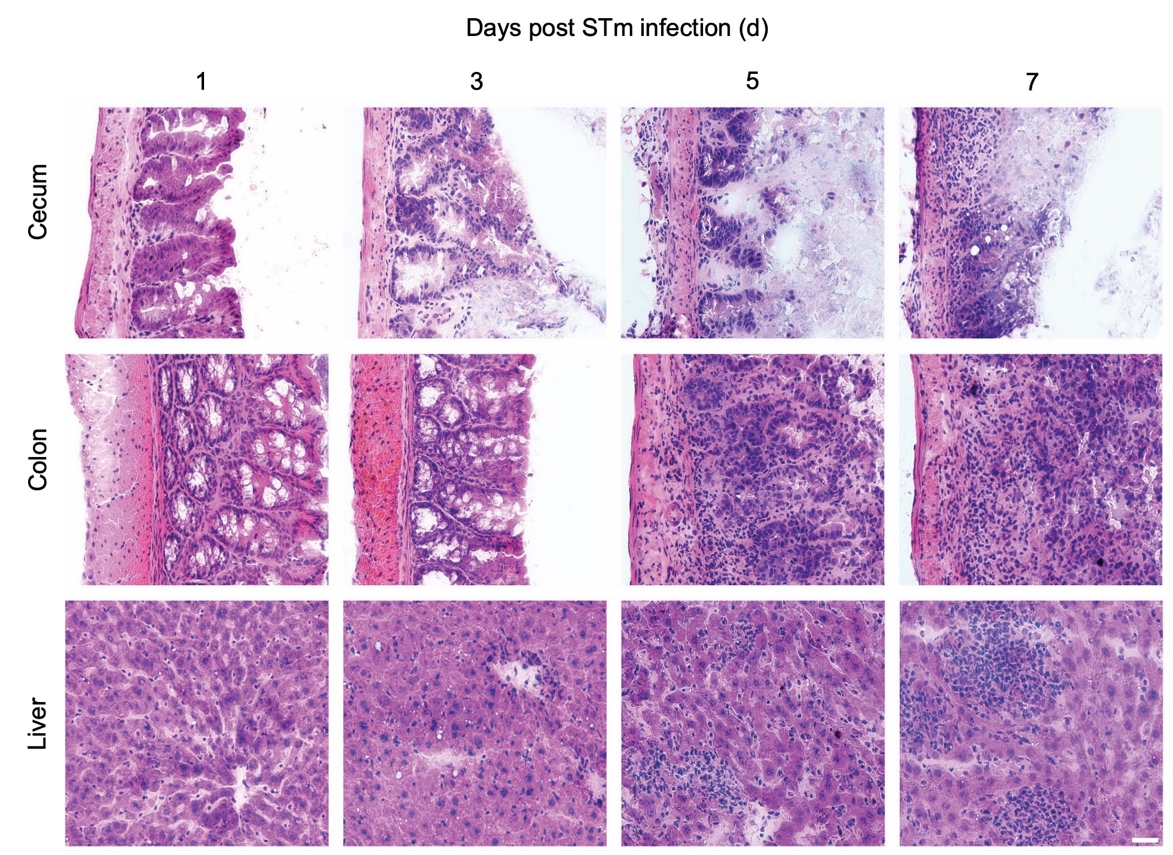
**

**Figure S1. STm destroys the intestinal epithelial structures, related to Figure 2.**

Representative H&E-stained micrographs of cecum, colon and liver tissue sections after STm infection. Scale bar, 25 μm.


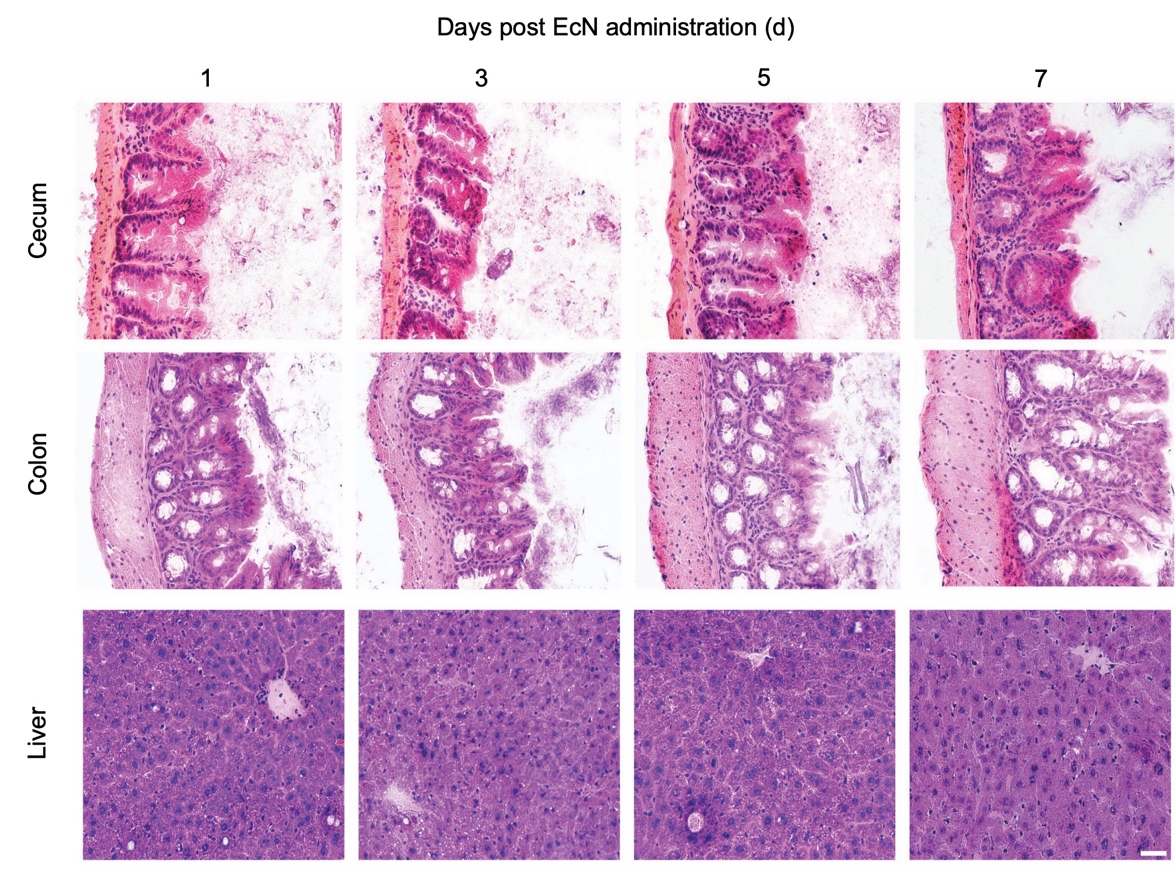


**Figure S2. The treatment of EcN maintains intestinal epithelial integrity, related to Figure 2.** Representative H&E-stained micrographs of cecum, colon and liver tissue sections after EcN treatment. Scale bar, 25 μm.


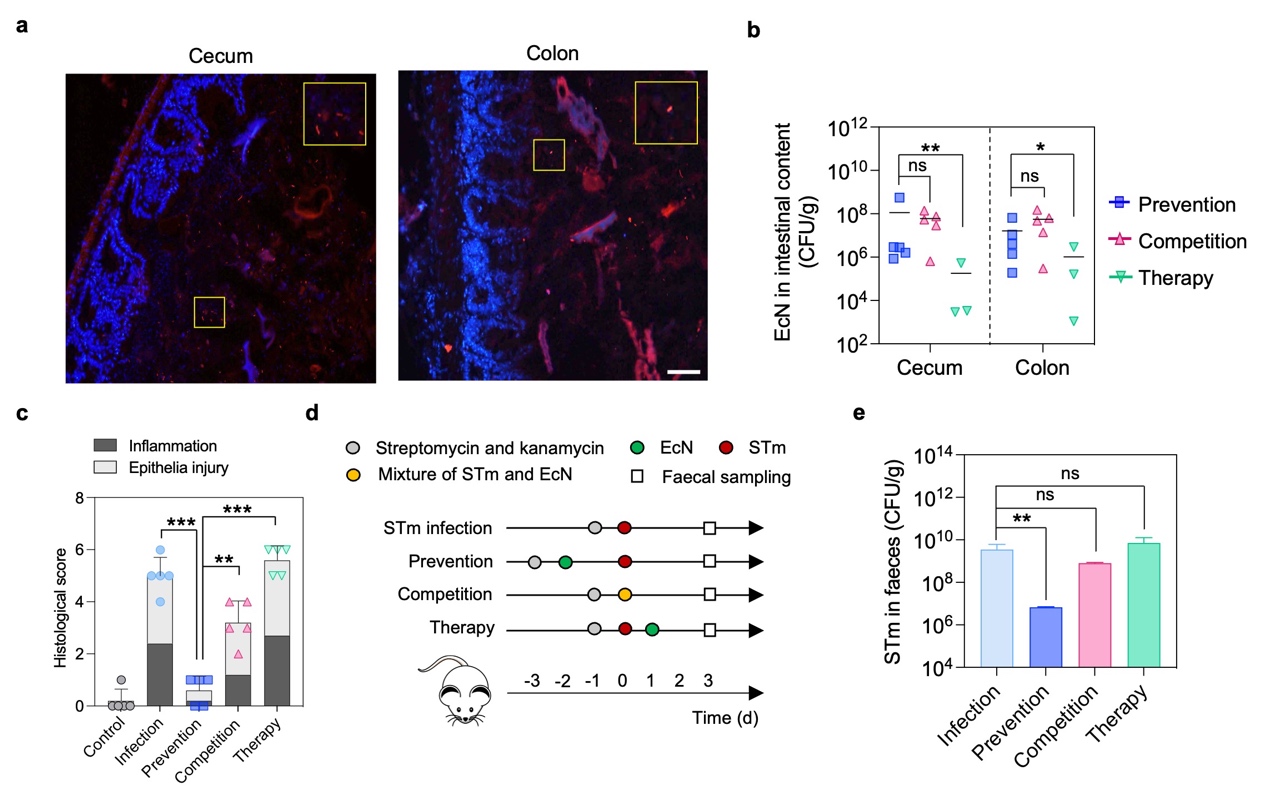


**Figure S3. EcN stabilizes in gut and attenuates STm colonization in the prevention group, related to Figure 2. a**, Representative fluorescence micrographs, showing the location and amount of EcN (red) in cecum and colon tissue frozen sections on day 7 after EcN treatment. Up right insets showed the enlarged images of the boxed areas. Scale bar, 50 μm. **b**, Amounts of EcN in the cecum and colon contents in each experimental group on day 7 after STm infection. **c**, Pathology scores of H&E-stained tissue sections from each experimental group on day 7 after STm infection (*n* = 5). **d**, Experimental scheme for examining effects of three EcN intervention in a short term. **e**, Comparison of STm loads in faeces samples on day 3 after STm infection in **d** (*n* = 5). Data (**c**,**e**) are presented as mean ± SD. Significance was calculated by unpaired t-test. **P* < 0.05, ***P* < 0.01, and ****P* < 0.001; ns, not significant.


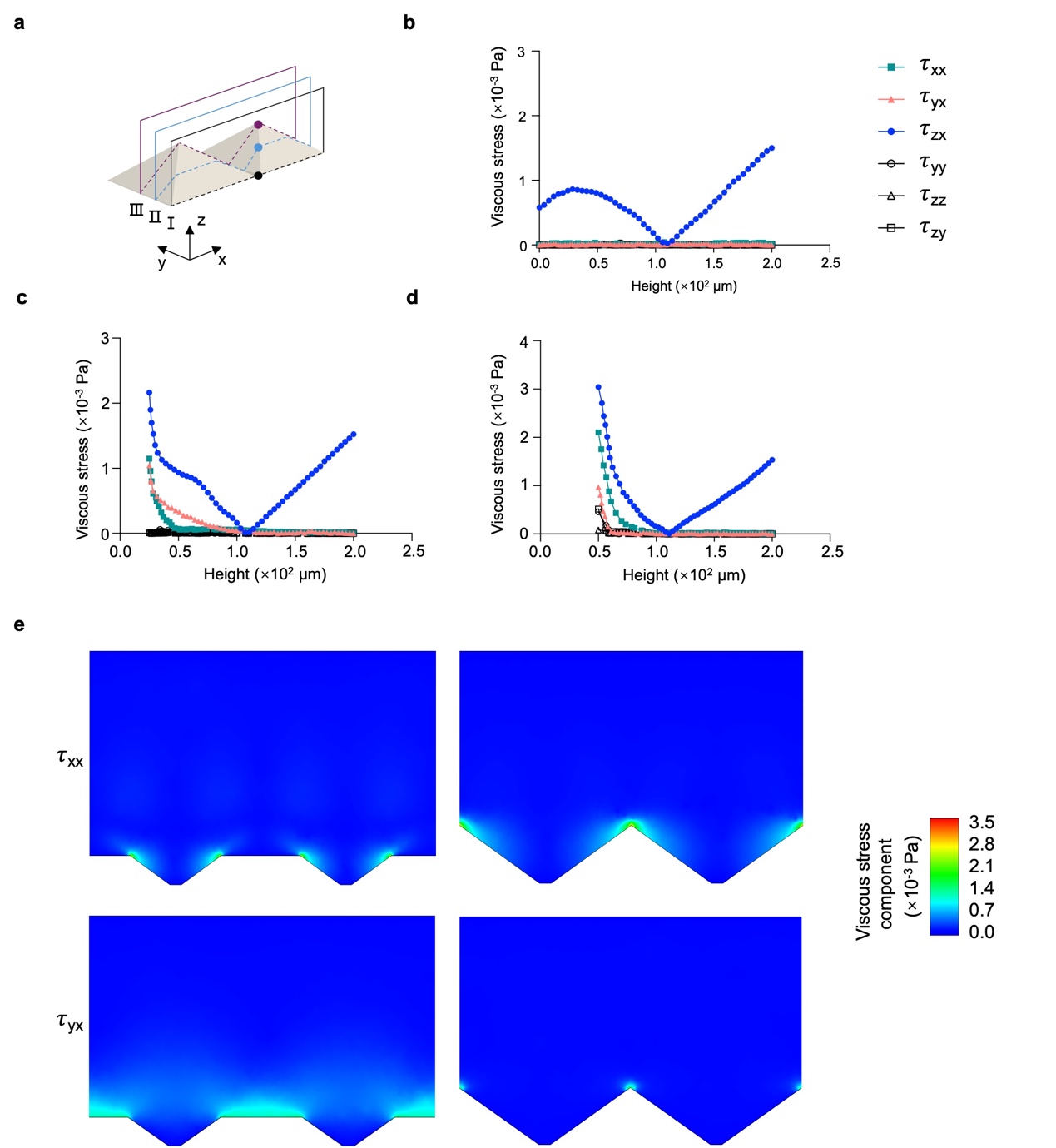


**Figure S4. The viscous stress by CFD simulations, related to Figure 3. a**, A schematic of the micropyramidal structure. **b**, Profiles of the viscous stress components along the z-axis at h = 0 µm (black dot in **a**), which is at the bottom of the crypt. **c**, Profiles of the viscous stress components along the z-axis at h = 25 µm (blue dot in **a**), which is in the middle of the villi-crypt. **d**, Profiles of the viscous stress components along the z-axis at h = 50 µm (purple dot in **a**), which is at the tip of the villus. **e**, Visualization of the viscous-stress components, *τ*_xx_ and *τ*_yx_, in the xz planes (II, III) designated with blue and purple lines in **a**.


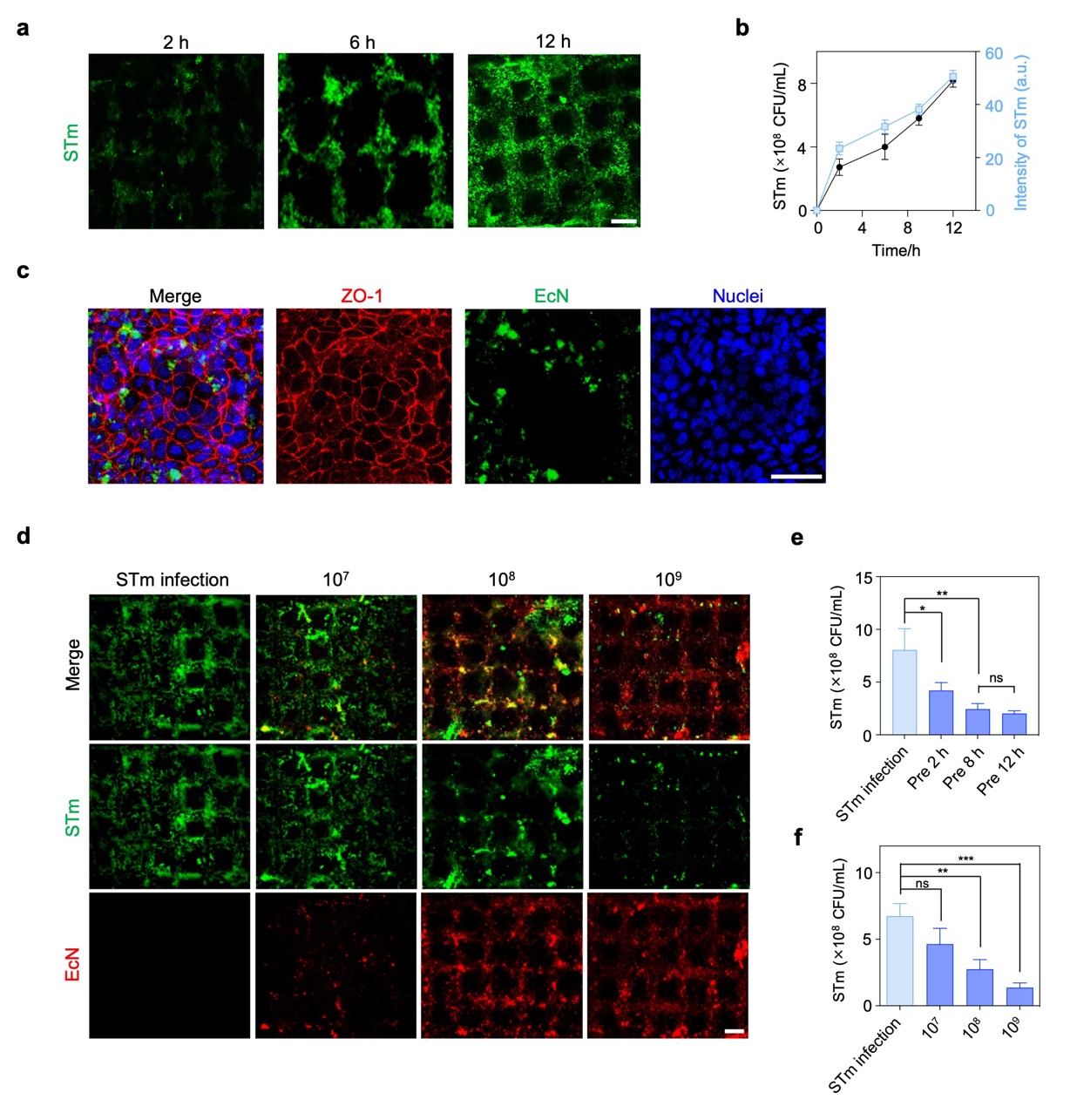


**Figure S5. Different effects caused by the treatment dosage and time in the preventive strategy, related to Figure 4. a**, Representative z-stack fluorescent confocal images of the GFP-labeled STm colonization (green) over time in the gut-on-chip. Scale bar, 100 μm. **b**, The growth curve of STm in the gut-on-chip. **c**, Immunofluorescence staining of ZO-1 (red) in cells co-cultured with EcN (10^9^ CFU/mL, green) for 12 h. Scale bar, 50 μm. **d**, Representative fluorescent confocal images, showing the distribution of STm (green) and EcN (red) under the prevention at different dosages of EcN (10^7^, 10^8^, 10^9^ CFU/mL). Scale bar, 100 μm. **e**, STm counting CFU in the gut-on-chip for different EcN pre-colonized time (2 h, 8 h and 12 h) at 10^9^ CFU/mL dosage of EcN. **f**, STm counting CFU in the gut-on-chip pre-colonized for 8 h by EcN at different dosages (10^7^, 10^8^ and 10^9^ CFU/mL). Data (**b**,**e**,**f**) are presented as mean ± SD (*n* = 3). Significance was calculated by unpaired t-test. **P* < 0.05, ***P* < 0.01, and ****P* < 0.001; ns, not significant.


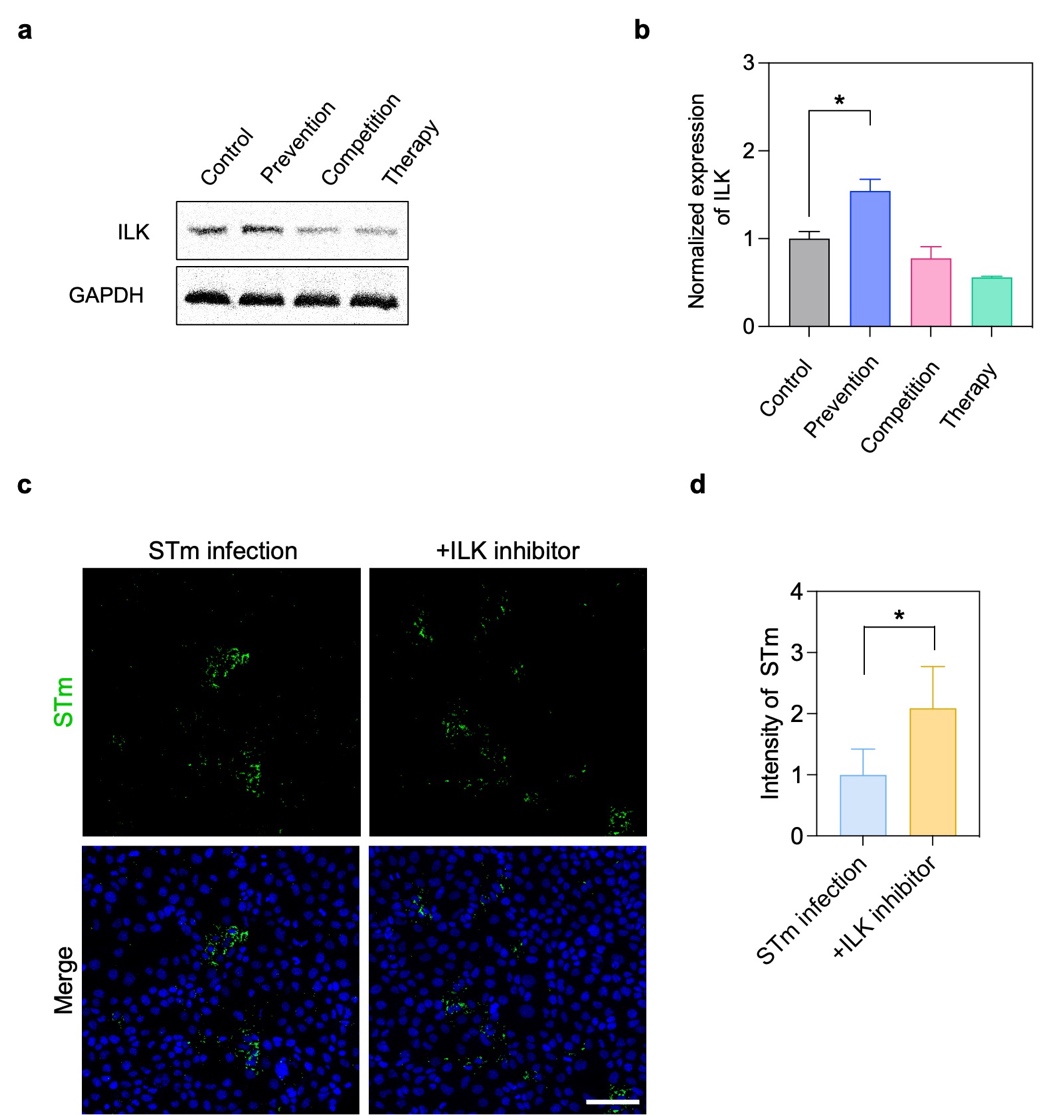


**Figure S6. The intracellular STm increases by the inhibition of ILK, related to Figure 5. a**, Immunoblotting of ILK in three EcN intervention strategies. GAPDH was the loading control. **b**, Fold changes of ILK expression determined by densitometry of protein bands (*n* = 3). **c**, Representative fluorescent confocal images, showing the distribution of intracellular STm without and with the addition of ILK inhibitor in a 6-well culture plate. Scale bar, 50 μm. **d**, Quantification of GFP fluorescence intensity of the intracellular STm in the presence of ILK inhibitor in (**c**) (*n* = 10). The fluorescence intensity of STm infection group was normalized to one. Data are presented as mean ± SD. Significance was calculated by unpaired t-test. **P* < 0.05.


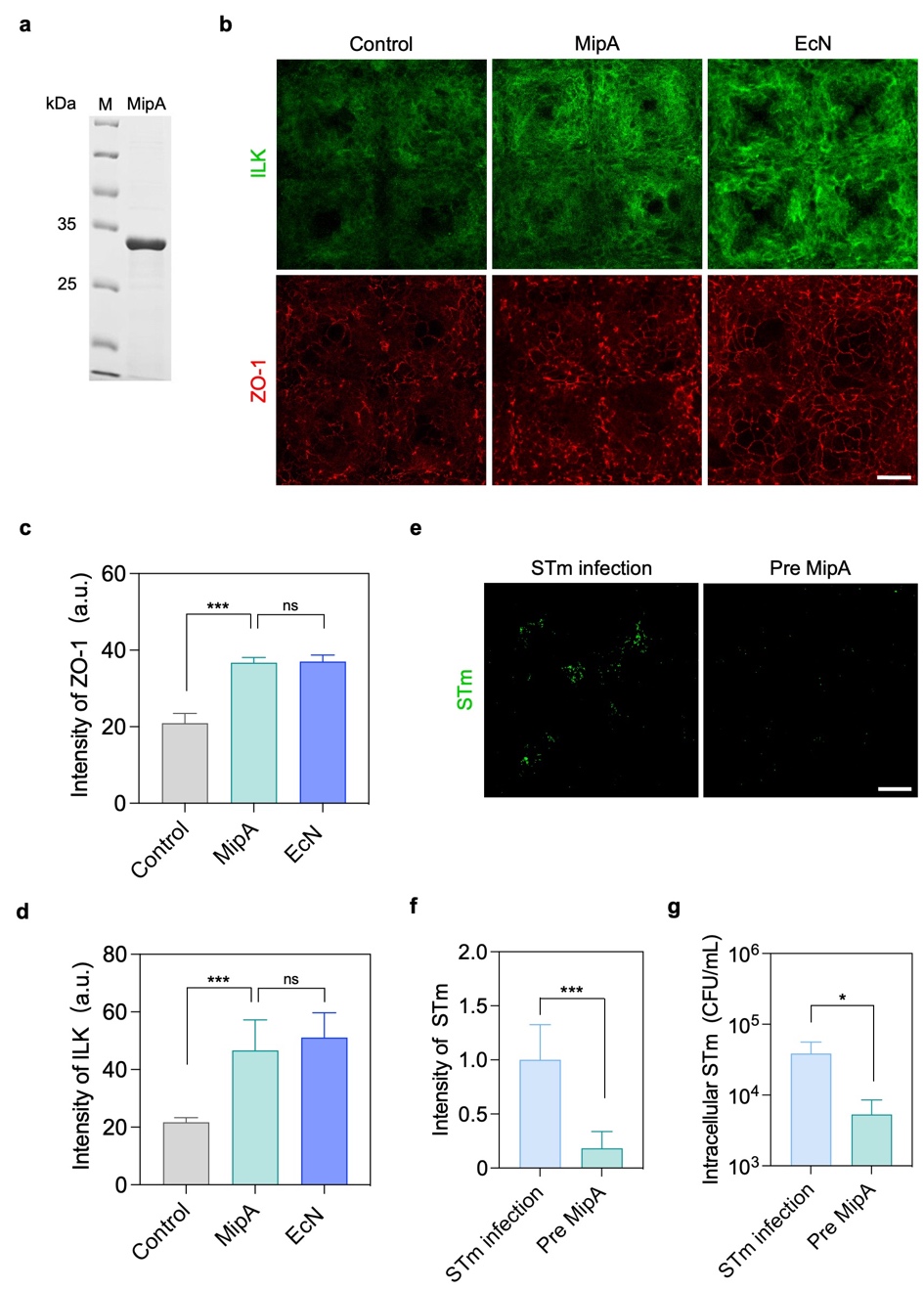


**Figure S7. MipA induces the ILK and ZO-1 expression levels, related to Figure 6. a**, Coomassie brilliant blue staining of MipA protein. **b**, Representative immunofluorescence images of ILK and tight junction protein ZO-1 in the gut-on-chip treated with purified MipA and EcN, respectively. Scale bar, 50 μm. **c**,**d**, Quantification of the fluorescence intensity of ZO-1 and ILK as the conditions in (**b**) (*n* = 10). **e**, Representative fluorescent confocal images, showing the distribution of intracellular STm with the pre-treatment of MipA in a 6-well culture plate. Scale bar, 50 μm. **f**, Quantification of GFP fluorescence intensity of the intracellular STm in (**e**) (*n* = 10). The fluorescence intensity of STm infection group was normalized to one. **g**, Quantification of intracellular STm counting CFU (*n* = 3). Data are presented as mean ± SD. Significance was calculated by unpaired t-test. **P* < 0.05, ****P* < 0.001; ns, not significant.


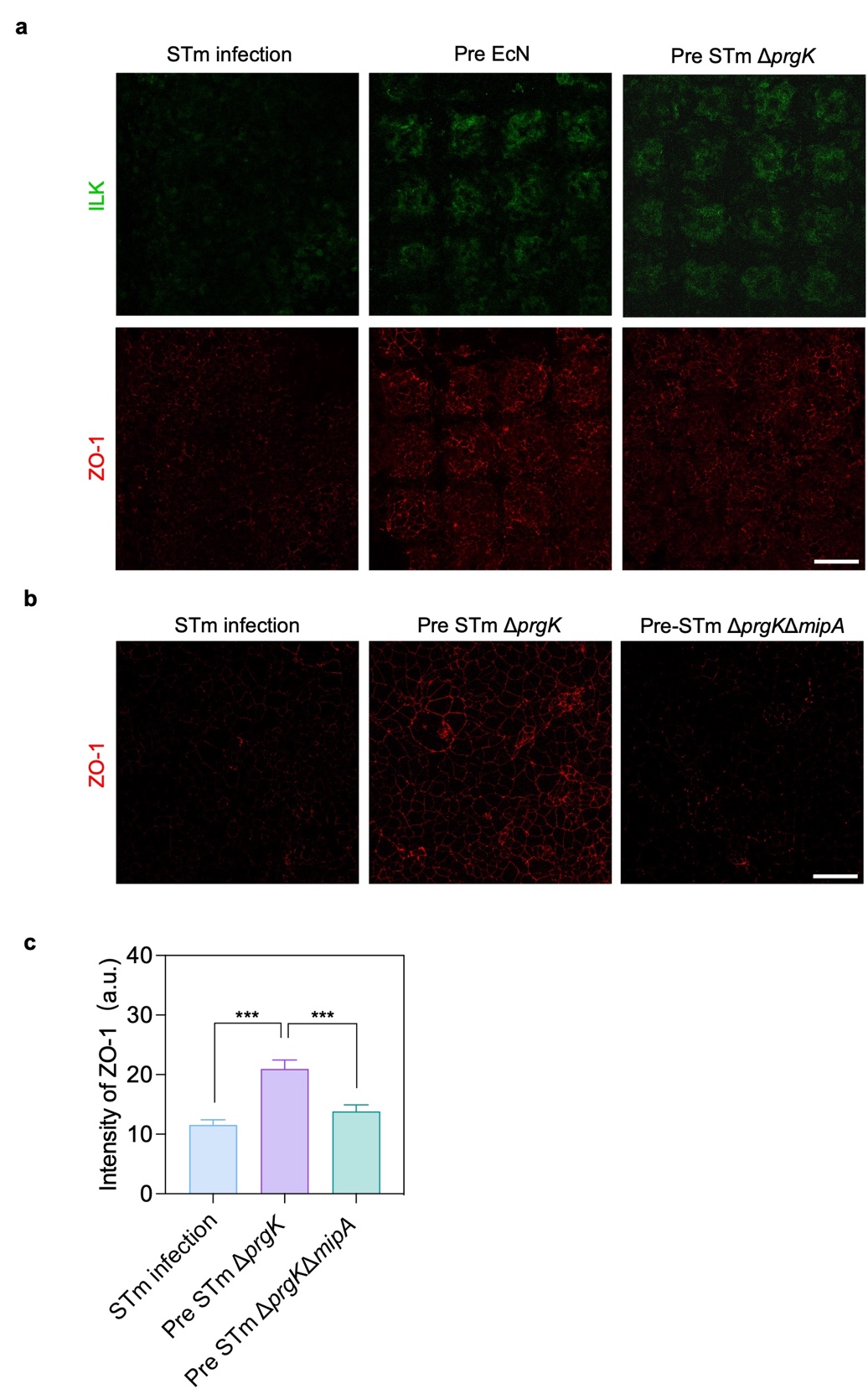


**Figure S8. Non-invasive STm Δ*prgK* strain prevents STm infection, related to Figure 6. a**, Representative immunofluorescence images of ILK and tight junction protein ZO-1 in the gut-on-chip. Scale bar, 100 μm. **b**, Representative immunofluorescence images of tight junction protein ZO-1 in intestinal cells cultured in a 6-well plate. Scale bar, 50 μm. **c**, Quantification of the fluorescence intensity of ZO-1 as the conditions in (**b**) (*n* = 10). Data are presented as mean ± SD. Significance was calculated by unpaired t-test. ****P* < 0.001.
